# Risk factors for undergoing surgery in patients with newly diagnosed open-angle glaucoma

**DOI:** 10.1038/s41598-022-09832-3

**Published:** 2022-04-05

**Authors:** Seung Jae Lee, Sang Ah Lee, Seungyeon Lee, Hyoung Won Bae, Chan Yun Kim, Gong Je Seong, Jong Woon Park, Kwanghyun Lee

**Affiliations:** 1 Institute of Vision Research, Department of Ophthalmology, Yonsei University College of Medicine, Seoul, South Korea; 2 Department of Ophthalmology, National Health Insurance Service Ilsan Hospital, 100 Ilsan-ro, Ilsandong-gu, Goyang, 10444 Korea; 3 Big Data Strategy Department, National Health Insurance Service, Wonju-si, Gangwon-do South Korea

**Keywords:** Glaucoma, Risk factors

## Abstract

Despite the clinical importance of glaucoma surgery, studies on its prevalence and risk factors are limited. We analyzed a database comprising approximately 1,000,000 Korean residents to investigate the prevalence and risk factors for undergoing glaucoma surgery within 5 years of diagnosis with open-angle glaucoma. Of the 4,303 patients evaluated, 226 (5.3%) underwent glaucoma surgery. Factors associated with the likelihood of glaucoma surgery included the use of two or more eye drops (odds ratio [OR], 30.30; 95% confidence interval [CI], 10.95–83.84), intake of oral carbonic anhydrase inhibitor (OR, 1.79; 95% CI, 1.23–2.61), age > 55 years (55–65 years: OR, 1.71; 95% CI, 1.06–2.76; > 65 years: OR 1.72; 95% CI, 1.10–2.70), female sex (OR, 1.46; 95% CI, 1.10–1.94), middle- and high-income (OR, 2.36; 95% CI, 1.30–4.28, OR, 1.86; 95% CI, 1.03–3.35, respectively), and metropolitan residence (OR, 1.61; 95% CI, 1.14–2.26). Our nomogram for predicting the likelihood of glaucoma surgery showed an acceptable result. In conclusion, older age, female sex, and the intensity of intraocular pressure lowering treatment increased the likelihood of undergoing glaucoma surgery. Our findings indicated that a lower socioeconomic status may forestall receiving this necessary surgery, which requires further attention.

## Introduction

Glaucoma is a progressive optic neuropathy characterized by optic disc changes and retinal ganglion cell loss, which can lead to irreversible loss of vision^1^. Various neuroprotective interventions are being studied to slow the progression of glaucoma, and certain medicines, like as citicoline and coenzyme q10^2,3^, have shown some potential. Although the most effective treatment for glaucoma currently is to lower intraocular pressure (IOP) with eye drops, when IOP is not sufficiently controlled or when eye drops cannot be used in certain conditions, such as in those with an allergy or poor adherence, surgery is required.

In the clinical setting, ophthalmologists are frequently asked about the possibility of vision loss due to glaucoma. Much research has been conducted on this topic, which has shown that higher IOP, older age, advanced visual field loss at the time of diagnosis, and residence in a developing country are risk factors for blindness^4–6^. Another question is whether the patient would need to undergo glaucoma surgery in the future. Despite its importance, studies on the prevalence and risk factors for glaucoma surgery are limited^7^. Considering that surgical treatment is performed as the last step to lower IOP, research related to this can provide information about glaucoma patients whose IOP was not adequately controlled with medical treatment. One recent study analyzed nationwide claim data and reported that factors, such as allergy-related comorbidities, were associated with undergoing trabeculectomy^7^. However, because that study compared a glaucoma surgery cohort and a non-glaucoma surgery cohort, the factors associated with undergoing surgery may differ from those of patients newly diagnosed with glaucoma.

In this study, we investigated risk factors leading patients with open-angle glaucoma (OAG) to undergo glaucoma surgery within 5 years of diagnosis. Data from only one or several hospitals may show a bias, since various factors, such as doctor’s preference, hospital size, and patient status (socioeconomic status, residential area) could affect the surgical decision. Thus, to avoid this bias, we analyzed nationwide claim data, the National Health Insurance Service-National Sample Cohort 2002–2015 (NHIS-NSC 2002–2015). We also investigated the prevalence and types of glaucoma surgery and presented a nomogram to predict a patient’s likelihood of undergoing surgery.

## Results

**Figure 1:**
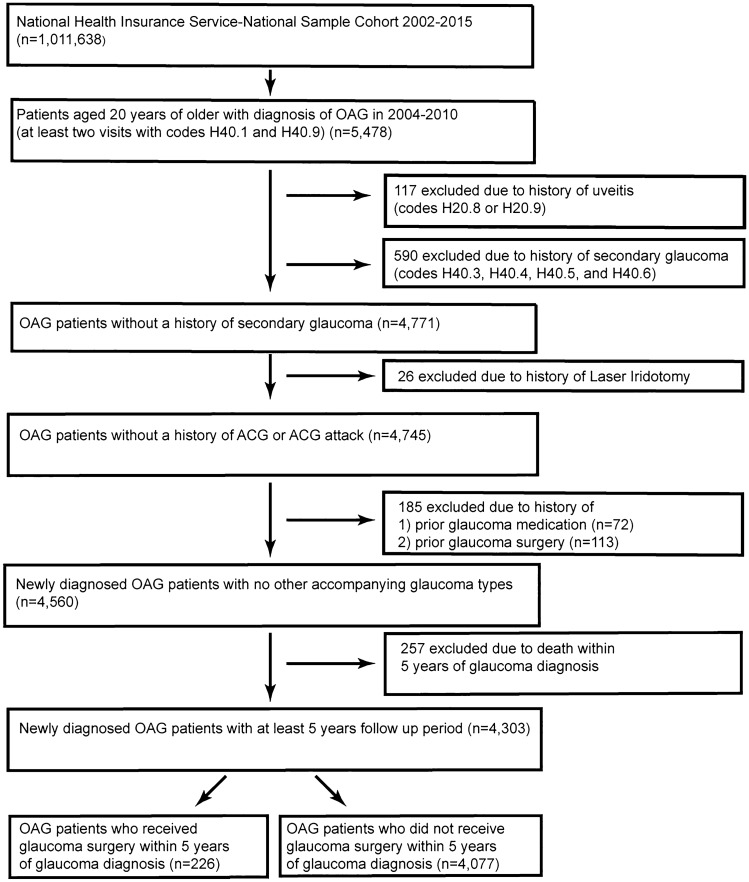
Flow diagram of study population selection. OAG: open-angle glaucoma, ACG: angle-closure glaucoma.

General characteristics of the study population are described in Table 1. Among 4303 patients newly diagnosed with OAG, 226 (5.3%) patients received glaucoma surgery within five years (Fig. 1). Age, use and number of glaucoma medications (eye drops), oral carbonic anhydrase inhibitor (CAI) intake, and residential area were statistically different between patients who received glaucoma surgery and those who did not. The results were the same when only the population using one or more eye drops were analyzed (supplemental Table 1).

**Table 1:** General characteristics of study population.

Variables	Total		Glaucoma operation				*P*-value
			No		Yes		
	*N*	(%)	*N*	(%)	*N*	(%)	
**Prescribed glaucoma eyedrops**							** < 0.001**
None	817	19.0	813	99.5	4	0.5	
PG	1296	30.1	1243	95.9	53	4.1	
Non-PG	1508	35.1	1440	95.5	68	4.5	
Two or more	682	15.9	581	85.2	101	14.8	
**Oral CAI intake**							** < 0.001**
No	3974	92.4	3796	95.5	178	4.5	
Yes	329	7.7	281	85.4	48	14.6	
**Age group**							**0.005**
< 45	782	18.2	751	96.0	31	4.0	
45–54	775	18.0	749	96.7	26	3.4	
55–64	1049	24.4	986	94.0	63	6.0	
65 +	1697	39.4	1591	93.8	106	6.3	
**Sex**							0.091
Men	1996	46.4	1904	95.4	92	4.6	
Women	2307	53.6	2173	94.2	134	5.8	
**Income**							0.088
Medical aids	386	9.0	372	96.4	14	3.6	
Low	707	16.4	672	95.1	35	5.0	
Middle	1265	29.4	1183	93.5	82	6.5	
High	1945	45.2	1850	95.1	95	4.9	
**Residential area**							**0.012**
Capital area	1993	46.3	1907	95.7	86	4.3	
Metropolitan area	974	22.6	907	93.1	67	6.9	
Rural area	1336	31.1	1263	94.5	73	5.5	
**Disability**							0.123
No	3836	89.2	3627	94.6	209	5.5	
Yes	467	10.9	450	96.4	17	3.6	
**Hypertension Hx**							0.966
No	3680	85.5	3486	94.7	194	5.3	
Yes	623	14.5	591	94.9	32	5.1	
**Diabetes Hx**							0.191
No	3484	81.0	3293	94.5	191	5.5	
Yes	819	19.0	784	95.7	35	4.3	
**Charlson Comorbidity Index**							0.950
0	1948	45.3	1844	94.7	104	5.3	
1	1451	33.7	1373	94.6	78	5.4	
2	642	14.9	611	95.2	31	4.8	
3 +	262	6.1	249	95.0	13	5.0	
**Total**	4303	100.0	4077	94.8	226	5.3	

PG: Prostaglandin analogue; CAI: Carbonic anhydrase inhibitor; Hx: History, *P-values were calculated using Chi-square test. Indicated in bold type, *P* < 0.05 indicates statistical significance.

Regarding the procedures, 154 (68.1%) patients underwent trabeculectomy, followed by cyclophotocoagulation (39 patients, 17.3%), drainage device implantation (15 patients, 6.6%), trabeculotomy (15 patients, 6.6%), and nonpenetrating surgery (3 patients, 1.3%).

The results of logistic regression are displayed in Table 2. Patients using two or more eye drops had an odds ratio (OR) of 30.30 of undergoing surgery compared to the patients with no medication. Patients older than 55 years, who were women, had a middle/high income, or who were residents in a metropolitan area had a higher OR of receiving glaucoma surgery. When the population using more than one eye drop was analyzed, the OR to undergo glaucoma surgery was significantly higher in patients aged greater than 55 years, women, residents in a metropolitan area, and those with a middle income (supplemental Table 2).

**Table 2:** Results of multiple logistic regression.

	Simple Logistic regression				Multiple logistic regression			
	Odds Ratio	95%	CI	*P*-value	Adjusted OR	95%	CI	*P*-value
**Prescribed glaucoma eyedrops**								
None	1.00				1.00			
PG	8.67	3.12	24.03	** < 0.001**	8.69	3.12	24.16	** < 0.001**
Non-PG	9.60	3.49	26.40	** < 0.001**	8.78	3.18	24.28	** < 0.001**
Two or more	35.33	12.93	96.49	** < 0.001**	30.30	10.95	83.84	** < 0.001**
**Oral CAI intake**								
No	1.00				1.00			
Yes	3.64	2.59	5.12	** < 0.001**	1.79	1.23	2.61	**0.003**
**Age group**								
< 45	1.19	0.70	2.02	0.523	1.28	0.75	2.21	0.367
45–54	1.00				1.00			
55–64	1.84	1.15	2.94	**0.010**	1.71	1.06	2.76	**0.027**
65 +	1.92	1.24	2.97	**0.004**	1.72	1.10	2.70	**0.019**
**Sex**								
Men	1.00				1.00			
Women	1.28	0.97	1.68	0.079	1.46	1.10	1.94	**0.009**
**Income**								
Medical aids	1.00				1.00			
Low	1.38	0.74	2.61	0.314	1.81	0.94	3.46	0.075
Middle	1.84	1.03	3.29	**0.039**	2.36	1.30	4.28	**0.005**
High	1.36	0.77	2.42	0.287	1.86	1.03	3.35	**0.038**
**Residential area**								
Capital area	1.00				1.00			
Metropolitan area	1.64	1.18	2.28	**0.003**	1.61	1.14	2.26	**0.006**
Rural area	1.28	0.93	1.77	0.128	1.13	0.81	1.58	0.459
**Disability**								
No	1.00							
Yes	0.66	0.40	1.09	0.101				
**Hypertension Hx**								
No	1.00							
Yes	0.97	0.66	1.43	0.890				
**Diabetes Hx**								
No	1.00							
Yes	0.77	0.53	1.11	0.164				
**Charlson Comorbidity Index**								
0	1.00							
1	1.01	0.75	1.36	0.962				
2	0.90	0.60	1.36	0.614				
3 +	0.93	0.51	1.67	0.798				

CI: confidence interval; OR: odds ratio; PG: Prostaglandin analogue; CAI: Carbonic anhydrase inhibitor; Hx: History, *P-values were calculated using logistic regression. Indicated in bold type, *P* < 0.05 indicates statistical significance.

**Figure 2:**
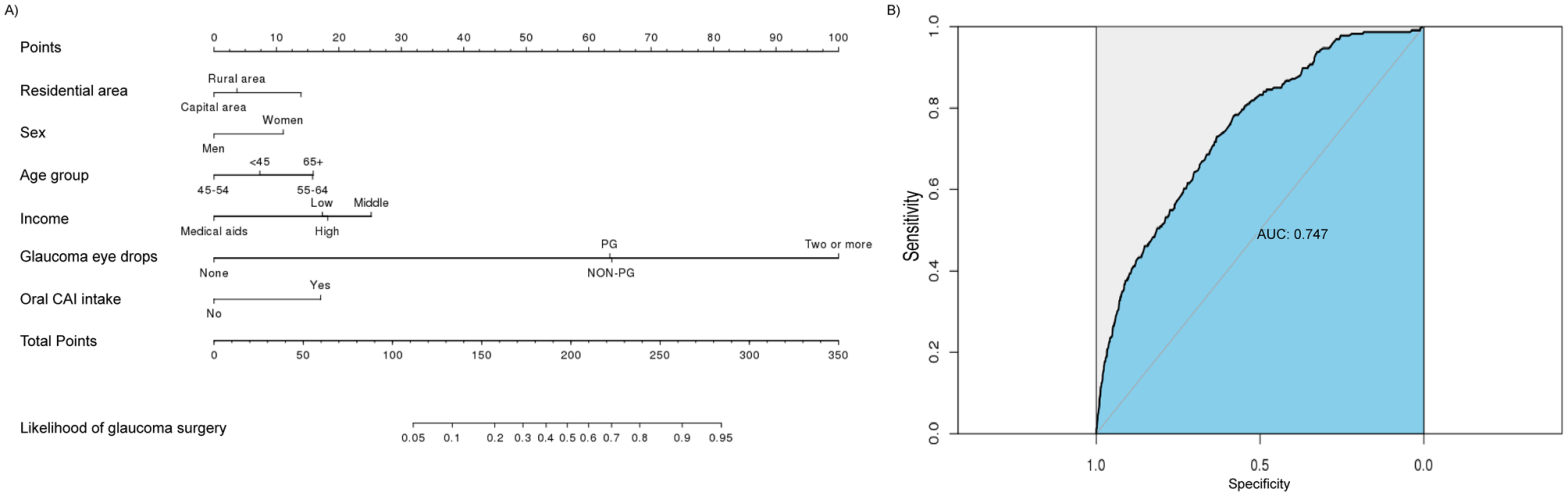
Nomogram and its receiver operating characteristics (ROC) curve for predicting the likelihood for newly diagnosed open-angle glaucoma patients to undergo glaucoma surgery. (**A**) Nomogram from multiple logistic regression model. Find the position of each variable on the corresponding axis and draw a line to the ‘Points’ scale (top axis) to determine the number of points for each variable. Sum the points for all the variables together and draw a line from the ‘Total points’ axis to determine the likelihood of undergoing glaucoma surgery at the bottom. (**B**) ROC curve and its diagnostic performance. AUC: area under the curve.

Based on the results of multiple logistic regression, we used residential area, sex, age, income, glaucoma medication, and oral CAI intake to construct the nomogram to predict the likelihood of undergoing glaucoma surgery (Fig. 2A). The area under the curve (AUC) of the model was 0.747 (Fig. 2B, supplemental Fig. 1).

Analysis of the associations between the number of patients’ visits in five years and the patients’ demographics was performed as well. The average numbers of patient visits were statistically different by residential area (*P* < 0.001) and income (*P* < 0.001), but not by sex (*P* = 0.317, supplemental table).

## Discussion

In this study, we analyzed the prevalence of surgical treatment in newly diagnosed OAG patients and risk factors for undergoing surgery using nationwide claim data. Those using a larger number of glaucoma eye drops, requiring oral CAI intake, with older age, female sex, higher income, or who were residents of a metropolitan area were more likely to undergo glaucoma surgery.

There are few previous studies on the rate of undergoing glaucoma surgery. According to our results, there was 5.3% chance that a patient with OAG would receive surgical treatment within five years of their first diagnosis. A retrospective study using the United States’ claims database reported that 4.2% of patients with open-angle glaucoma received surgery within 48 months of diagnosis^8^. Considering that our study’s observation period was longer, at five years, the rates are considered to be similar. These results suggest that the rate of glaucoma surgery required is not significantly different between Korea, where normal-tension glaucoma is more common^9^, and the United States, where normal-tension glaucoma is relatively rare^10^. In other words, it can be inferred that the proportion of patients requiring surgery due to insufficient control of IOP is similar regardless of the level of baseline IOP or types of OAG.

Our results showed that patients using two or more eye drops or taking oral CAI were more likely to undergo surgery. In addition, whether or not glaucoma eye drops were used and the number of glaucoma eye drops used were the most significant variables in the nomogram. These results could be explained by the fact that glaucoma surgery is performed when IOP is not controlled or the target IOP is not reached, even with medical treatment. In addition, in our study, the proportion of those who were prescribed two or more eye drops was approximately 19.5% of eye drop-using patients, consistent with a previous study that reported about 76% of glaucoma patients were treated by monotherapy^11^. In other words, these results suggest that about 20% of all glaucoma patients do not have adequate IOP control with monotherapy alone.

An unexpected finding of our study was that 19.0% (817) of the patients did not receive glaucoma medications. Some patients with mild ocular hypertension or glaucoma suspects may have been included in this study, since their diagnostic codes were set to "primary open-angle glaucoma" and "unspecified glaucoma." A previous study using the US health insurance claims database to investigate treatment patterns in patients with newly diagnosed OAG found that 1052 (17.0%) of 6172 individuals diagnosed with OAG did not receive treatment^8^, which is similar to our findings. This error could be difficult to avoid while using the claims database for study. Another unexpected finding was that four patients in the group without prescribed drugs underwent glaucoma surgery. However, because the type of eye drops was defined in our study as medications prescribed within one year of diagnosis, it is understandable that part of the non-prescription population received glaucoma surgery as the disease worsened.

The risk of undergoing glaucoma surgery increased significantly in those over 55 years of age. In previous studies, age was found to be an important risk factor for glaucoma progression. In the Early Manifest Glaucoma Trial, older age at baseline was one of the main risk factors for both the development and progression of glaucoma^12^. Additionally, in a meta-analysis of a population-based study, the OR for primary OAG was 1.73 (95% CI, 1.63–1.82) for each decade increase in age beyond 40 years^13^. There may be three possible explanations for the higher surgical rate in older glaucoma patients: (1) IOP may not be well controlled in older patients, (2) glaucoma may progress more rapidly in older patients, and/or (3) glaucoma may have already advanced in older patients at the time of diagnosis. Unfortunately, nationwide claim data do not have information about each patient’s IOP, glaucoma progression rate, or glaucoma stage at the time of diagnosis, so we were unable to analyze whether the aforementioned hypotheses actually influenced our results. However, the significantly higher rate of glaucoma surgery in the older patients, even after adjusting for glaucoma medication use, suggests that uncontrolled IOP is not a major cause of our results. Nevertheless, since these results did not directly analyze IOP, further research is needed.

The rate of undergoing glaucoma surgery was higher among females. According to the United States database, the 2002–2005 National Health Interview Survey, women had a higher prevalence of self-reported visual impairment due to glaucoma (OR: 1.20; 95% CI, 0.99–1.45)^14^. In addition, female sex has been reported as a risk factor of glaucoma progression^15,16^. Considering that there is no clear association of the development of primary OAG and female sex^10,17^, this result is quite interesting. It is also speculated that female sex hormones have a protective effect on the optic nerve, and that postmenopausal women lose this protective effect, leading to rapid glaucoma progression^18^. At the same time, the higher prevalence of glaucoma surgery in females may be due to poor access to medical care or good adherence to treatment. According to a review of the United Healthcare database, women were 24% less likely to receive treatment for glaucoma^19^. However, in this study, there was no difference in the average visits between men and women, so the lack of accessibility is not considered to be the reason for the higher prevalence of glaucoma surgery. On the other hand, in a previous study conducted in Korea, it was reported that women had good adherence to glaucoma treatment^11^. Considering this, the higher prevalence of glaucoma surgery in women may be because some male glaucoma patients were not adherent and refused surgery even when needed.

Among socioeconomic factors, income and residential area showed a significant association with the rate of undergoing glaucoma surgery. Patients receiving medical aid had lower rates of glaucoma surgery than middle- and high-income individuals. These results suggest that there may be some populations who do not receive surgical treatment due to economic conditions. On the other hand, our results on the association between residential area and glaucoma surgery rates were different from our expectation. A previous study that compared the characteristics of patients with primary OAG in rural and urban areas reported a higher percentage of uncontrolled IOP and more structural damage in the rural area^20^. The percentage of unilateral and bilateral blindness was also higher in the rural area (52.2%, 34.1% in rural, 32.9%, 17.5% in urban, respectively) compared with the urban area^20^. In contrast to the pervious study, our study reported that metropolitan areas had the highest OR for glaucoma surgery. Since the average number of visits of the metropolitan area was similar to that of the rural area and higher than that of the capital area, the higher odds of glaucoma surgery in the metropolitan area do not seem to be induced by poor accessibility in the residential area. Therefore, additional research is needed to find the cause of higher rate of glaucoma surgery in the metropolitan area.

This study had several limitations. First, due to the nature of nationwide claim data, clinical information, including disease severity and IOP, could not be analyzed. Therefore, we could not investigate the association between IOP and the rate of undergoing glaucoma surgery and only estimated the status IOP by the number of eye drops. Furthermore, because individual medical records of patients could not be inspected, it was impossible to rule out the possibility that some individuals with OAG diagnostic codes were indeed glaucoma suspects. Therefore, we analyzed only the population that used one or more eye drops to decrease this inaccuracy. Second, due to its rapid advancements, micro invasive glaucoma surgery (MIGS) should be taken into consideration. However, since we used cohort data from 2004 to 2010, data for MIGS could not be included and analyzed. Third, the diagnosis of glaucoma may be inaccurate because glaucoma was defined using only the diagnostic code and drug prescription code. For example, there may be patients who come to the hospital for an accurate diagnosis of glaucoma because they are suspected of having glaucoma during a medical examination. During this process, if the patient’s diagnostic code is glaucoma, the incidence of glaucoma could be overestimated. To reduce such risks, we set a washout period of two years and analyzed patients who visited ophthalmologist more than twice with a diagnosis of glaucoma.

In summary, older age, female sex, more eye drops use, and oral CAI intake were associated with an increased likelihood of undergoing glaucoma surgery. Our results that residential area and income also correlated with the likelihood of undergoing surgery suggest that there could be some populations who need surgery but are unable to do so due to their socioeconomic status. In addition, this study presented a nomogram that can calculate the need for future surgical treatment in patients newly diagnosed with OAG, which we hope will help to predict the prognosis of patients with glaucoma.

## Methods

### Statement of ethics

This study was approved and written informed consent was waived due to the retrospective cohort study design by the Institutional Review Board of National Health Insurance Service Ilsan Hospital (2020–03-038) and followed the tenets of the Declaration of Helsinki.

### Database

The Korean National Health Insurance Service (KNHIS), as the only public medical insurance institution operated by the ministry of Health and welfare in Korea, has been managing national health insurance since 1989. The NHIS-NSC 2002–2015 included approximately 1 million Korean residents in its initial 2002 cohort. For research purposes, the KNHIS used a systemic sampling method and created this data set of the Korean population (2.2%). This database includes all medical information related to insurance claims. The KNHIS and medical providers exchanged all cost-related healthcare information using the electronic codes of Korean electronic data interchange (KEDI) (e.g., the KEDI code for “Visual Field Examination (Automated)” is E6691, and for “Cosopt; Merck and Co., Inc.” is 655,500,390 (E09060211)). Therefore, this database provides detailed information on procedures and prescription drugs, as well as diagnostic codes and personal information.

### Study sample

OAG was defined when all the following criteria were met: (1) patients who visited ophthalmologist at least twice a year received a diagnosis of “primary open-angle glaucoma” and “unspecified glaucoma” (Korean Classification of Diseases [KCD], H40.1 and H40.9) from 2004 to 2010, (2) patients without a history of visiting the ophthalmologist received a diagnosis of iridocyclitis (H20.8 and H20.9) after being diagnosed with glaucoma, (3) patients without a history of secondary glaucoma (H40.3–40.6), (4) patients without a treatment history of laser iridotomy (surgical code 5030). The following surgical codes were for nonpenetrating filtering surgery (S5040), iridectomy (S5041), filtering surgery (S5042), trabeculectomy (S5043), cyclophotocoagulation (S5044), cyclocryosurgery (S5045), and glaucoma implant surgery (S5049) in patients with glaucoma. To yield the incidence of glaucoma from 2004 to 2010, we set up a wash-out period of two years and excluded the patients who were diagnosed with glaucoma from 2002 to 2003.

### Variables

To investigate the relationship between eye drops and glaucoma surgery, the types of eye drops used in the year following the glaucoma diagnosis were analyzed. The type of eye drops prescribed was determined to be prescribed within one year of glaucoma diagnosis. When a patient switched from using one eye drop to another, the latter eye drop was selected and analyzed. Age was grouped into four categories: < 45, 45–54, 54–64, and > 64 years. Sex was categorized as male or female. Household income was grouped into four categories (medical aids, low, middle, and high) and residential area was categorized into three groups (capital, metropolitan, and rural area). Disability was grouped into no/yes groups as well as the groups defined by the presence or absence of hypertension and diabetes. The Charlson comorbidity index (with the exception of acquired immune deficiency syndrome, which was not included to protect privacy) was evaluated, including a total of 15 comorbidities based on the KCD in 2002.

### Statistical analysis and nomogram building

Descriptive statistics of the study population are presented as frequency and percentage for categorical variables. The difference in demographics between the glaucoma patients undergoing surgery and the glaucoma patients without a history of surgery were compared with the Chi-square test (or Fisher’s exact test if cell expectations were less than five). To identify the factors associated with glaucoma surgery, simple and multiple logistic regression analyses were conducted. We also analyzed whether the average number of patient visits differed by sex, residential area, or income using the Chi-square test. Statistical analyses were performed using SAS System for Windows, version 9.4 (SAS Institute Inc., Cary, NC, USA). All P values were two sided and were considered statistically significant when less than 0.05.

We proposed a nomogram to calculate the likelihood that glaucoma patients would undergo surgery. A nomogram is composed of graphical lines of risk factors, points, total points, and risk of event. The length of each risk factor’s line was made to reflect the regression coefficient estimated by multiple logistic regression. We selected significant risk factors from multiple logistic regression. The estimated effect of each variable was ranked based on the estimated beta coefficient in multiple logistic regression. The AUC of receiver operating characteristic analysis was used to evaluate the discriminative performance of the nomogram. A calibration curve was used to measure the nomogram calibration. Internal validation was performed from 200 additional bootstrap samples to decrease the overfit bias.

## Supplementary Information

Supplementary Information.

## Data Availability

This study was used NHIS-NSC data (NHIS-2020–2-105) made by National Health Insurance Service. The authors declare no conflict of interest with NHIS. This study was approved by the Institutional Review Board of National Health Insurance Service Ilsan Hospital (approval number: NHIMC 2020-03-038). The data that support the findings of this study are available from NHIS, but restrictions apply to the availability of these data, which were used under license for the current study; therefore, these are not publicly available. Data are, however, available from the authors upon reasonable request and with permission of NHIS.
