# Supplementary material for: Risk factors for undergoing surgery in patients with newly diagnosed open-angle glaucoma
Source: Sci Rep. 2022 Apr 5;12:5661. doi: 10.1038/s41598-022-09832-3 (PMC8983768; doi:10.1038/s41598-022-09832-3)
Supplement: Supplementary file 1 — Supplementary Information. [file 41598_2022_9832_MOESM1_ESM.pdf]

Supplemental figure 1.

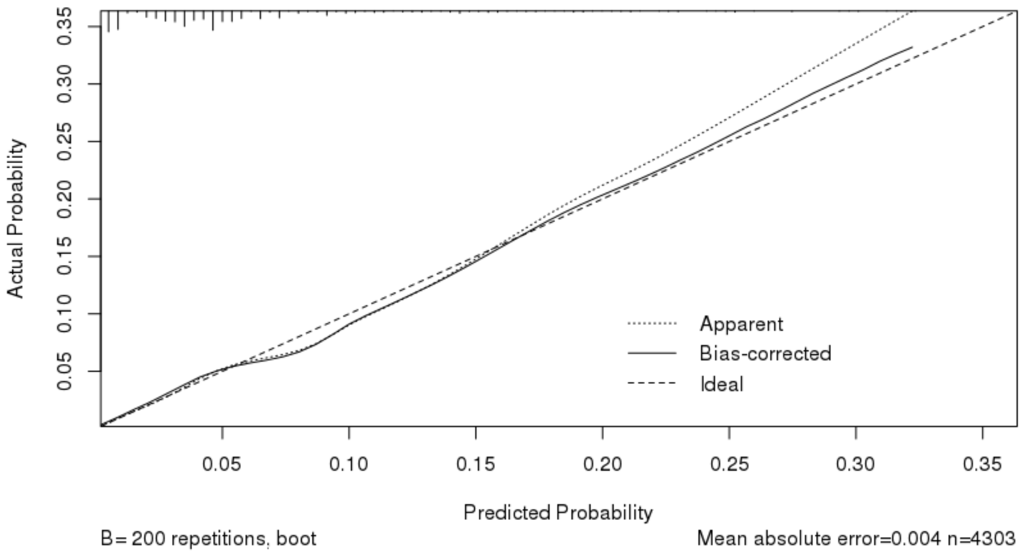

Calibration plot of logistic model. The calibration curve revealed that the bias-corrected lines, which represent the performance of the bootstrap-corrected nomogram

Supplemental Tables

Table 1. General characteristics of study population

Variables	Total		Glaucoma operation				P-value
			No		Yes		
	N	(%)	N	(%)	N	(%)	
**Prescribed glaucoma eyedrops**							**<.0001**
PG	1,296	37.2	1,243	95.9	53	4.1	
Non-PG	1,508	43.3	1,440	95.5	68	4.5	
Two or more	682	19.6	581	85.2	101	14.8	
**Oral CAI intake**							**<.0001**
No	3,157	90.6	2,983	94.5	174	5.5	
Yes	329	9.4	281	85.4	48	14.6	
**Age group**							**0.006**
<45	619	17.8	589	95.2	30	4.8	
45–54	622	17.8	597	96.0	25	4.0	
55–64	840	24.1	778	92.6	62	7.4	
65+	1,405	40.3	1,300	92.5	105	7.5	
**Sex**							0.063
Men	1,631	46.8	1,541	94.5	90	5.5	
Women	1,855	53.2	1,723	92.9	132	7.1	
**Income**							0.056
Medical aids	313	9.0	299	95.5	14	4.5	
Low	578	16.6	544	94.1	34	5.9	
Middle	1,021	29.3	939	92.0	82	8.0	
High	1,574	45.2	1,482	94.2	92	5.8	
**Residential area**							**0.018**
Capital area	1,606	46.1	1,522	94.8	84	5.2	
Metropolitan area	795	22.8	730	91.8	65	8.2	
Rural area	1,085	31.1	1,012	93.3	73	6.7	
**Disability**							0.127
No	3,121	89.5	2,915	93.4	206	6.6	
Yes	365	10.5	349	95.6	16	4.4	
**Hypertension Hx**							>0.999
No	2,989	85.7	2,799	93.6	190	6.4	
Yes	497	14.3	465	93.6	32	6.4	
**Diabetes Hx**							0.179
No	2,825	81.0	2,637	93.3	188	6.7	
Yes	661	19.0	627	94.9	34	5.1	
**Charlson Comorbidity Index**							0.951
0	1,572	45.1	1,470	93.5	102	6.5	
1	1,174	33.7	1,098	93.5	76	6.5	
2	534	15.3	503	94.2	31	5.8	
3+	206	5.9	193	93.7	13	6.3	
**Total**	3,486	100	3,264	93.6	222	6.4	

PG: Prostaglandin analogue; CAI: Carbonic anhydrase inhibitor; Hx: History, * P-values were calculated using Chi-square test. Indicated in bold type, P <0.05 indicates statistical significance.

Table 2. Results of multiple logistic regression

	Simple Logistic regression				Multiple logistic regression			
	Odds Ratio	95% CI		P-value	Adjusted OR	95% CI		P-value
**Prescribed glaucoma eyedrops**								
PG	1.00				1.00			
Non-PG	1.11	0.77	1.6	0.586	1.13	0.78	1.63	0.516
Two or more	4.08	2.88	5.77	**<.0001**	4.10	2.74	6.14	**<0.001**
**Oral CAI intake**								
No	1.00				1.00			
Yes	2.93	2.08	4.12	**<.0001**	0.94	0.61	1.44	0.779
**Age group**								
<45	1.22	0.71	2.09	0.408	1.16	0.67	2.01	0.604
45-54	1.00				1.00			
55-64	1.9	1.18	3.06	**0.008**	1.78	1.10	2.90	**0.019**
65+	1.93	1.23	3.02	**0.004**	1.74	1.11	2.74	**0.017**
**Sex**								
Men	1.00				1.00			
Women	1.31	0.99	1.73	0.055	1.30	0.98	1.72	0.071
**Income**								
Medical aids	1.00				1.00			
Low	1.33	0.71	2.53	0.375	1.33	0.69	2.54	0.391
Middle	1.87	1.04	3.34	**0.036**	1.91	1.06	3.45	**0.032**
High	1.33	0.75	2.36	0.337	1.37	0.76	2.45	0.296
**Residential area**								
Capital area	1.00				1.00			
Metropolitan area	1.61	1.15	2.26	**0.005**	1.55	1.10	2.18	**0.013**
Rural area	1.31	0.95	1.81	0.105	1.33	0.95	1.84	0.093
**Disability**								
No	1.00							
Yes	0.65	0.39	1.09	0.103				
**Hypertension Hx**								
No	1.00							
Yes	1.01	0.69	1.49	0.945				
**Diabetes Hx**								
No	1.00							
Yes	0.76	0.52	1.11	0.153				
**Charlson Comorbidity Index**								
0	1.00							
1	1.00	0.73	1.36	0.987				
2	0.89	0.59	1.34	0.575				
3+	0.97	0.53	1.76	0.922				

CI: confidence interval; OR: odds ratio; PG: Prostaglandin analogue; CAI: Carbonic anhydrase inhibitor; Hx: History, * P-values were calculated using logistic regression. Indicated in bold type, P <0.05 indicates statistical significance.

Table 3. Average number of visits and residential area

Level of Residential Area	N	COUNT Mean	Std Dev	<.0001
Capital area	1993	13.24586	14.5231	
Metropolitan area	974	18.2423	20.01764	
Rural area	1336	17.58159	17.81648	

Table 4. Average number of visits and sex

Level of SEX	N	COUNT Mean	Std Dev	<0.3167
Men	1996	16.00351	17.04535	
Women	2307	15.48028	17.13302	

Table 5. Average number of visits and income

Level of INCOME	N	COUNT Mean	Std Dev	<.0001
Medical aids	386	21.79534	22.68673	
Low	707	14.89109	15.33685	
Middle	1265	15.26166	17.21261	
High	1945	15.12031	16.07633	
